# Cross-Corpus Speech Emotion Recognition Based on Multi-Task Learning and Subdomain Adaptation

**DOI:** 10.3390/e25010124

**Published:** 2023-01-07

**Authors:** Hongliang Fu, Zhihao Zhuang, Yang Wang, Chen Huang, Wenzhuo Duan

**Affiliations:** 1 College of Information Science and Engineering, Henan University of Technology, Zhengzhou 450001, China; 2 Henan Engineering Laboratory of Grain IOT Technology, Henan University of Technology, Zhengzhou 450001, China; 3 Key Laboratory of Food Information Processing and Control, Ministry of Education, Henan University of Technology, Zhengzhou 450001, China

**Keywords:** speech emotion recognition, multi-task learning, subdomain adaptation, feature distribution

## Abstract

To solve the problem of feature distribution discrepancy in cross-corpus speech emotion recognition tasks, this paper proposed an emotion recognition model based on multi-task learning and subdomain adaptation, which alleviates the impact on emotion recognition. Existing methods have shortcomings in speech feature representation and cross-corpus feature distribution alignment. The proposed model uses a deep denoising auto-encoder as a shared feature extraction network for multi-task learning, and the fully connected layer and softmax layer are added before each recognition task as task-specific layers. Subsequently, the subdomain adaptation algorithm of emotion and gender features is added to the shared network to obtain the shared emotion features and gender features of the source domain and target domain, respectively. Multi-task learning effectively enhances the representation ability of features, a subdomain adaptive algorithm promotes the migrating ability of features and effectively alleviates the impact of feature distribution differences in emotional features. The average results of six cross-corpus speech emotion recognition experiments show that, compared with other models, the weighted average recall rate is increased by 1.89~10.07%, the experimental results verify the validity of the proposed model.

## 1. Introduction

Speech is a very valuable research object to realize intelligent interaction today. Through speech communication, human beings can not only obtain the speaker’s semantic information, but also perceive the speaker’s emotional state, gender, age and other paralinguistic content [1]. In the middle of the 20th century, human–computer interaction (HCI) systems mainly conveyed instructions to computers through the mouse and keyboard, and did not have the ability to perceive speech emotional information. In order to improve the intelligence of a computer and meet the comfortable and convenient needs of users, it is particularly important to make the computer have the speech-emotional information perception ability like human beings. In this context, researchers began to explore the emotional information processing of speech.

Speech Emotion Recognition (SER) first began using acoustic statistical features to classify emotions [2] in the 1980s, these acoustic features are still widely used in speech analysis [3,4]. With the rapid development of artificial intelligence in the 21st century, speech emotion recognition technology has been widely used in various fields, including call quality detection in a customer service center, speech assistants and auxiliary diagnoses. Therefore, SER has very important practical application research value.

In real application scenarios, different corpora have different recording environments, personnel gender, age distribution and languages, resulting in great variations in feature distribution among different corpora, which makes it difficult for models trained based on a single corpus to achieve good recognition results on new speech signal [5]. Speech emotion recognition also has some limitations in other aspects. For example, in the case of strong background noise, emotional information is difficult to be effectively recognized. Therefore, many scholars try to supplement it with other aspects, including facial emotion recognition [6,7,8] and physiological signal emotion recognition [9,10].

In order to further enhance the generalization of the speech emotion recognition model, the main contributions of this work are summarized as follows:

1. The proposed method uses multi-task learning to help the network extract speech features, which is more robust than the features obtained only using emotional recognition tasks.
2. A subdomain transfer learning method is proposed, which can reduce the negative transfer in the whole local adaptation process more than the global adaptation method.
3. In the ablation experiment and the evaluation compared with other algorithms, the proposed method has achieved performance leadership in most cross-corpus schemes.

## 2. Related Work

At present, the recognition rate of speech emotion recognition has reached the level of human recognition, but this can only be achieved under the condition of acoustic laboratory and some specific emotion corpus. When the training data and test data come from different corpora, the model performance often suffers a serious decline. Many researchers propose cross-corpus algorithms to solve the data discrepancy to improve the model performance.

Deng et al. [11] used unsupervised learning methods of denoising auto-encoder and domain adaptive technology to solve the inherent difference between the training set and the test set. Huang et al. [12] proposed a new feature transfer method based on PCANet to learn the emotional features of unlabeled data by measuring the distribution offset between training data and test data. Zong et al. [13] proposed a domain adaptive least squares regression model. The least squares regression model was trained by adding regularization constraints of source domain data and a group of target domain data to the objective function to improve cross-corpus recognition performance. In addition, the subspace learning algorithm has also achieved satisfactory results in the cross-corpus SER. For example, Liu et al. [14] proposed a domain adaptive subspace learning method to learn the projection matrix and convert the speech signal from the original feature space to the label subspace; Song et al. [15] proposed a transfer linear subspace learning framework, and used the nearest neighbor graph algorithm to measure the similarity between different corpora, so as to achieve cross-corpus speech emotion recognition research; Luo et al. [16] extracted the source domain data and target domain data to obtain the shared subspace feature representation and two independent feature representations, and used the orthogonal constraint method to eliminate the redundancy of shared features and independent features, while minimizing the difference between the conditional distribution and marginal distribution of the source domain and target domain in the shared subspace. Finally, they achieved high recognition rates in 30 sets of cross-corpus emotion recognition experiments. In addition, the combination of deep learning and domain adaptation to solve cross-corpus speech emotion recognition problems has gradually become a new research focus. For example, Liu et al. [17] used the depth convolution neural network and the maximum mean discrepancy (MMD) to perform feature migration and achieve cross-corpus speech emotion recognition.

Therefore, the influencing factors of cross-corpus SER system performance can be summarized as follows:

1. To obtain the emotional information with strong representation ability in speech feature. Human speech contains a variety of paralinguistic information in addition to semantic information, such as mood, gender, emotion, but the ideal speech emotional feature should be independent of the speaker, semantics, language and other objective factors, and reflect emotional information as effectively as possible, which puts forward higher requirements for the generalization of emotional features of the cross-corpus SER system.
2. To effectively measure the distribution discrepancy of features. In cross-corpus SER research, researchers mostly use the emotion feature measurement criteria based on the global feature area [12,13,17], and only measure the distance between two emotion vector matrices representing the source domain and target domain, ignoring the differences of different emotion features in the field, which may lead to the confused transfer of similar emotion information, such as happy and surprise, anger and disgust, which is not conducive to the subsequent emotion classification.

## 3. Model Framework

**Figure 1:**
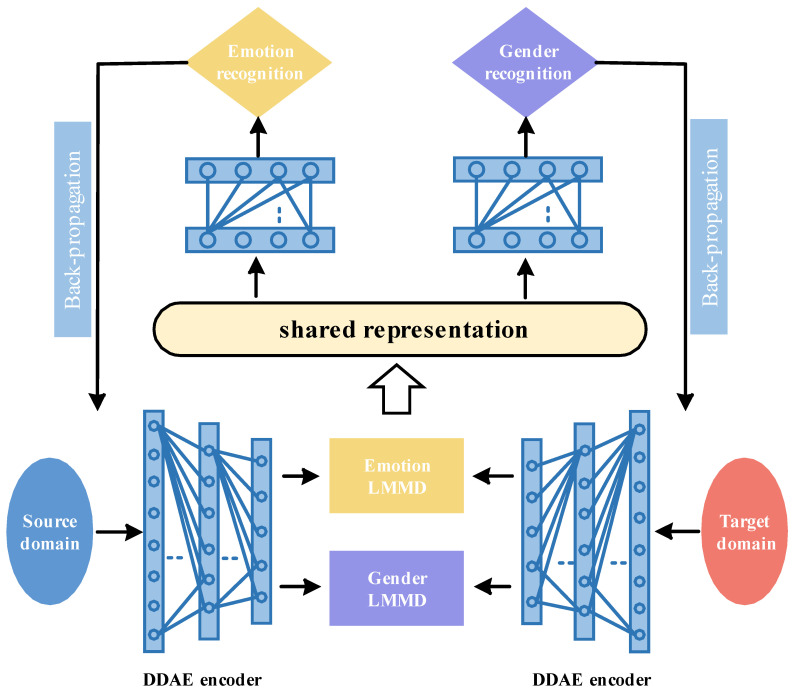
Overall Framework of Multi-task Learning and Subdomain Adaptive Model.

Multi-task learning can improve the generalization of the main task recognition performance. This chapter introduces a cross-corpus SER model based on Multi-task learning and subdomain adaptation (MTLSA), as shown in Figure 1. First, it is confirmed that the main recognition task of MTLSA is emotion recognition, while the auxiliary recognition task is gender recognition. Secondly, in the aspect of feature processing, the model MTLSA in this chapter uses the deep denoising auto-encoder (DDAE) network as the task-sharing network. On this basis, task-specific layers with attribute dependency are added, so that when the network learns the shared features, it allows each task-specific layer to optimize its own attribute parameters to improve performance. Then, in the low dimensional emotional features output by the DDAE code, the whole region is divided into emotional subdomain space and gender subdomain space according to emotional labels and gender labels, and the subdomain adaptation algorithm based on the local maximum mean discrepancy (LMMD) [18] is used to reduce the feature distribution distance between the source domain and target domain. Finally, the cross entropy loss calculation is performed using the emotion label and gender label information of the source domain, and the MTLSA is constrained by the feature reconstruction loss and feature distribution distance measurement loss. The MTLSA multi-task learning module and subdomain adaptation will be described in detail in Section 3.1 and Section 3.2, and the MTLSA training and recognition process will be described in Section 3.3.

### 3.1. Multi-Task Learning

In the cross-corpus SER research, in order to further reduce the discrepancy in the distribution of emotional features and improve the generalization of the system, the multi-task learning mechanism is introduced to eliminate the emotional differences caused by gender factors, so as to learn more common emotional information between different fields. In this section, MTLSA performs feature matching under the multi-task learning mechanism based on hyper-parameter sharing. The sharing network of the emotion recognition task and gender recognition task is DDAE. It has been verified that the reconstructed features can effectively compress feature dimensions and remove feature redundancy. On this basis, the model adds noise to the DAE and builds a DDAE network to extract common emotional features from the source domain and target domain to enhance system robustness.

The sample features of the source domain are given as follows: $X_{S}=[x_{1}^{S},\cdots ,x_{n_{S}}^{S}]\in R^{d\times n_{S}}$, the emotional category label of the source domain sample is $Y_{S}=[y_{1},\cdots ,y_{n_{S}}]\in R^{C\times n_{S}}$, the gender category label of the source domain sample is $Y_{G}=[y_{1},\cdots ,y_{n_{S}}]\in R^{2\times n_{S}}$, and the sample features of the target domain is $X_{T}=[x_{1}^{T},\cdots ,x_{n_{T}}^{T}]\in R^{d\times n_{T}}$. Among this, $n_{S}$ and $n_{T}$ represent the number of samples in the source domain and target domain, respectively, d represent the emotional feature dimension of each speech sample, and C represent the number of emotional categories. DDAE is used for redundant compression of speech features to obtain common emotional features with robustness and effective representation. First, add the noise with the normal distribution (mean value is 0, variance is 1) in the source domain $X_{S}$ and target domain $X_{T}$. Then, low-level features with noise are input into DDAE, and the source domain and target domain feature vectors decoded by DDAE are represented as ${\tilde{X}}_{S}$ and ${\tilde{X}}_{T}$, respectively. Therefore, the loss function of the DDAE network processing features includes the reconstruction loss function $L_{S}$ of $X_{S}$ and the reconstruction loss function $L_{T}$ of $X_{T}$, which are, respectively, expressed as:

(1) $$L_{S}=(X_{S},{\tilde{X}}_{S})=\sum_{i=1}^{n_{S}}{\left\|x_{i}^{S}-{\tilde{x}}_{i}^{S}\right\|}^{2}$$

(2) $$L_{T}=(X_{T},{\tilde{X}}_{T})=\sum_{i=1}^{n_{T}}{\left\|x_{i}^{T}-{\tilde{x}}_{i}^{T}\right\|}^{2}$$

The task-specific layer consists of two independent full connection layers, which input the results into the softmax layer and output the emotion labels. In the cross-corpus research based on domain adaptation, the main task emotion recognition and the auxiliary task gender recognition will use the source domain real label information and the source domain softmax prediction label to calculate the cross entropy as a loss function to constrain the parameter update of different tasks at the specific layer. The prediction probabilities of the emotion category and gender category of the source domain samples are expressed as $p_{i}^{S}=[p_{1}^{S},\cdots ,p_{n_{S}}^{S}]$ and $p_{i}^{G}=[p_{1}^{G},\cdots ,p_{n_{S}}^{G}]$, respectively, and the cross entropy is calculated with the ground truth, respectively, and the emotion classification loss function $L_{Y}$ and gender classification loss function $L_{G}$ of the source domain are obtained.

(3) $$L_{Y}(Y_{S},p_{i}^{S})=-\frac{1}{n_{S}}\sum_{i=1}^{n_{S}}\sum_{c=1}^{C}y_{i}^{S}\log(p_{i}^{S})$$

(4) $$L_{G}(Y_{G},p_{i}^{G})=\frac{1}{n_{S}}\sum_{i=1}^{n_{S}}-\left[y_{i}^{G}\cdot \log(p_{i}^{G})+(1-y_{i}^{G})\cdot log(1-p_{i}^{G})\right]$$

### 3.2. Subdomain Adaptation

**Figure 2:**
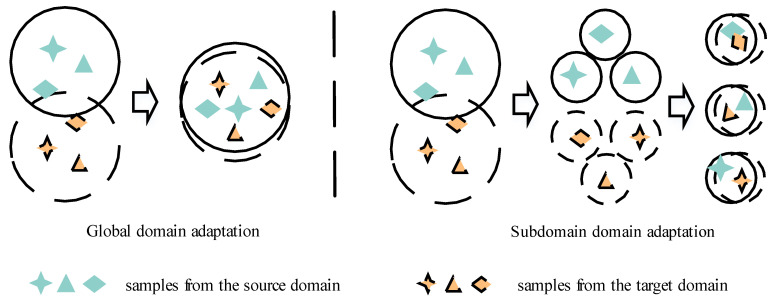
Differences between subdomain adaptation and global domain adaptation.

To learn common emotional information through gender recognition tasks by multi-task learning. At the same time, it uses a subdomain adaptive algorithm based on Local Maximum Mean Discrepancy (LMMD) to measure the feature distributions discrepancy between the source domain and the target domain, as shown in Figure 2, so as to reduce the emotional differences and gender differences in speech and improve the generalization of the system. The MTLSA model divides the low dimensional features output by the DDAE encoder into independent emotion subdomain space and gender subdomain space according to the emotion labels and gender labels of the source domain, and the emotion prediction label and gender prediction label of the target domain, so as to achieve accurate emotion feature alignment and gender feature alignment.

In the emotion subdomain space, the emotion features output by the source domain and target domain through the DDAE encoder are represented as ${X^{\prime }}_{S}=[{x^{\prime }}_{1}^{S},\cdots ,{x^{\prime }}_{n_{S}}^{S}]\in R^{d^{\prime }\times n_{S}}$ and ${X^{\prime }}_{T}=[{x^{\prime }}_{1}^{T},\cdots ,{x^{\prime }}_{n_{T}}^{T}]\in R^{d^{\prime }\times n_{T}}$, respectively, and the feature distribution is aligned through LMMD, and the measured distribution distance can be used as loss function $L_{DE}$ to continuously reduce during the training process.

(5) $$L_{DE}=\frac{1}{C}\sum_{c=1}^{C}{\left\|\frac{1}{n_{S}}\sum_{i=1}^{n_{S}}\mu_{i,c}^{S}\delta ({x^{\prime }}_{i}^{S})-\frac{1}{n_{T}}\sum_{i=1}^{n_{T}}\mu_{i,c}^{T}\delta ({x^{\prime }}_{i}^{T})\right\|}_{H}^{2}$$

Among them, H is the reproducing kernel hilbert space (RKHS), and δ(⋅) represents the kernel function that maps emotional features to RKHS. $\mu_{i,c}^{S}$ and $\mu_{i,c}^{T}$, respectively, represent the weight vectors of ${x^{\prime }}_{i}^{S}$ and ${x^{\prime }}_{i}^{T}$ belonging to the emotion category. The weight $\mu_{i,c}^{}$ of sample feature ${x^{\prime }}_{i}$ is calculated as $\mu_{i,c}=y_{i,c}/\sum_{(x_{i},y_{i})\in D}y_{i,c}$. It is worth noting that the emotional label $y_{i,c}^{S}$ of the sample features in the source domain is known, while the target domain cannot directly obtain $y_{i,c}^{T}$. Here, softmax outputs the sample feature probability of the target domain to generate the pseudo tag $y_{i,c}^{T}$.

In the gender subdomain space, the gender features of the source domain and target domain encoded by DDAE are ${X^{\prime }}_{SG}=[{x^{\prime }}_{1}^{SG},{x^{\prime }}_{2}^{SG},\cdots ,{x^{\prime }}_{n_{S}}^{SG}]\in R^{d^{\prime }\times n_{S}}$ and ${X^{\prime }}_{TG}=[{x^{\prime }}_{1}^{TG},{x^{\prime }}_{2}^{TG},\cdots ,{x^{\prime }}_{n_{S}}^{TG}]\in R^{d^{\prime }\times n_{T}}$, respectively. Similarly, gender features are aligned by LMMD, and the metric distance is expressed as $L_{DG}$.

(6) $$L_{DG}=\frac{1}{M}\sum_{m=1}^{M}{\left\|\frac{1}{n_{S}}\sum_{i=1}^{n_{S}}\beta_{i,M}^{S}\delta ({x^{\prime }}_{i}^{SG})-\frac{1}{n_{T}}\sum_{i=1}^{n_{T}}\beta_{i,M}^{T}\delta ({x^{\prime }}_{i}^{TG})\right\|}_{H}^{2}$$

Wherein, $\beta_{i,M}^{S}$ and $\beta_{i,M}^{T}$, respectively, represent the weight vectors of source domain feature ${x^{\prime }}_{i}^{SG}$ and target domain ${x^{\prime }}_{i}^{TG}$ that belong to the gender category M. M=2, like Formula (5), $y_{i,M}^{T}$ cannot be directly obtained. The target domain samples need to generate pseudo label information $y_{i,M}^{T}$ through softmax output.

### 3.3. Model Training and Identification

The total loss function of the MTLSA can be expressed as:

(7) $$L_{SUM}=a\cdot L_{S}+b\cdot L_{T}+c\cdot L_{Y}+d\cdot L_{G}+e\cdot L_{DE}+f\cdot L_{DG}$$

Among them, $\left\{L_{S},L_{T},L_{Y},L_{G},L_{DE},L_{DG}\right\}$ represents the reconstruction loss of source domain sample features, the reconstruction loss of target domain sample features, the emotional classification loss function of source domain sample features, the gender classification loss function of source domain sample features, the emotional feature distribution distance, and the gender feature distribution distance, respectively. {a,b,c,d,e,f} represents the loss weight coefficient of $\left\{L_{S},L_{T},L_{Y},L_{G},L_{DE},L_{DG}\right\}$, respectively, and the values of a+b+c+d+e+f=1 and {a,b,c,d,e,f} are determined through debugging.

In the recognition stage, the target domain samples are used as the test corpus, and the emotion features are extracted from the trained network. After the softmax layer outputs the prediction probability, the label information corresponding to the maximum probability value is selected as the sample recognition result, and the emotion labels of the target domain samples are finally output.

## 4. Experimental Setup

### 4.1. Corpus

In order to ensure the consistency of the experiment and the fairness of the evaluation of the experimental indicators, the proposed method uses the most widely used corpus for evaluation. Three public corpora, Berlin [19], eNTERFACE [20], and CASIA [21] are selected as the corpora of the experiment. Berlin is recorded by five male and five female actors simulating anger, boredom, disgust, fear, neutral and sad. eNTERFACE included 34 male and eight female subjects anger, disgust, fear, happy, sad and surprise. CASIA contains the anger, fear, happy, neutral, sad and surprise of two male and three female speakers. In order to carry out cross-corpus research, we selected the samples of source domain and target domain that come from different corpora, but the emotional labels of the two corpora are the same. Therefore, three samples of three corpora need to be reselected to meet the experimental requirements.

In terms of emotion recognition, the same emotions of Berlin and eNTERFACE are disgust, anger, sad, fear and happy, and the sample numbers are 375 and 1072, respectively. The same emotions of eNTERFACE and CASIA are surprise, anger, sad, fear and happy, and the sample numbers are 1072 and 1000, respectively. The same emotions of Berlin and CASIA are neutral, anger, sad, fear and happy, with 408 and 1000 samples selected, respectively.

In identifying gender, we need to make gender tags of three corpora. The samples of the material corpus used in the two identification tasks are exactly the same, only the label types are different. Among them, the number of male samples in Berlin and eNTERFACE is 159 and 885, respectively, and the number of female samples is 216 and 187, respectively; eNTERFACE and CASIA. The number of male samples in the library is 847 and 500, respectively, and the number of female samples is 225 and 500, respectively. The number of male samples in Berlin and CASIA is 187 and 500, respectively, and the number of female samples is 221 and 500, respectively. Table 1 summarizes the corpus information used for cross-corpus identification.

### 4.2. Extract Speech Features

This section uses the emotional feature set specified in the INTERSPEECH2010 emotional challenge as the speech of all emotional feature set. Based on 34 LLDs, 1428 dimensional features are obtained by using 21 statistical functions. Secondly, on the basis of LLDs and delta coefficients of four treble, 152 dimensional features are obtained by using 19 statistical functions. Then, add the start time and duration of the speech into it. Finally, a total of 1582 dimensional artificial statistical emotional feature set is obtained [22]. Use the openSMILE tool [23] to extract 1582 dimension features of three corpora in Table 1. In addition, these speech features need to be normalized before input network training to compress the eigenvalues in the (0, 1) range.

### 4.3. Experimental Scheme

Choose between two corpora randomly from the three corpora, and choose speech samples with the same emotion between the two corpora to design the experimental scheme, one of which is used as the source domain corpus, the other as the target domain corpus. Using the letters B, E and C to represent Berlin, eNTERFACE and CASIA, respectively, six cross-corpus speech emotion recognition experimental schemes are designed, which are E→B, B→E, E→C, C→E, B→C, C→B. Table 2 summarizes the source domain and target domain of different cross-corpus experimental schemes, as well as the cross-corpus identification tasks of each scheme.

In the six experimental schemes, the learning rate and batch size of MTLSA are set to 0.000001 and 100, respectively, the network optimizer and classifier use Adam and softmax, respectively, and the model is iteratively trained 300 times. In the training process, the weight coefficients {a, b, c, d, e, f} of the six loss functions of the model are [0.05, 0.05, 0.6, 0.1, 0.1, 0.1]. For DDAE, the sizes of hidden layer neuron nodes are 1200, 900, 256, 900 and 1200, respectively, where the encoding and decoding stages use the ELU function and Sigmoid function, respectively. In addition, each layer of DDAE adds a Batch Normal (BN) layer and a Dropout layer. For task-specific layers in multi-task learning, the hidden layer neuron node size is 256.

## 5. Analysis of Experimental Results

### 5.1. Analysis of Ablation Experiment

This section conducts ablation experiments to evaluate the effectiveness of different modules in MTLSA, and sets up two ablation models. (1) MTLSA_L indicates that the proposed model MTLSA only uses the LMMD algorithm for emotional feature distribution alignment and gender feature distribution alignment, and does not use multi-task learning; (2) MTLSA_M means that MTLSA only uses the multi-task learning framework to learn shared features, and does not use the LMMD algorithm for feature alignment. In the six cross-corpus experimental schemes, the experimental results of two ablation models and MTLSA are shown in Table 3.

From Table 3, it can be seen that the WAR of the proposed model MTLSA in this chapter is higher than those of other ablation models under the six schemes, indicating that it is an effective practice for MTLSA to combine multi-task learning with subdomain adaptive feature transfer. From the WAR of MTLSA_L and MTLSA_M, it can be seen that MTLSA only uses a deep denoising auto-encoder to extract common features, and on this basis, LMMD is used to measure the distribution distance of emotional features and gender feature distribution distance, and the system performance of using LMMD to measure the distribution distance of emotional features is poor, while the multi-task learning architecture is used to extract common features, and the use of auxiliary tasks to learn emotion-related information is beneficial to obtain more emotional features, effectively reducing the feature distribution distance between the source domain and the target domain. Multi-task learning and subdomain adaptation are both forms of transfer learning, and the fusion of the two can extract salient emotional features and effectively improve the generalization of the system.

### 5.2. Comparative Experimental Analysis

In this section, some state-of-the art cross-corpus SER models are used for comparison to evaluate the performance of MTLSA, including Transfer Sparse Discriminant Subspace Learning (TSDSL) [22], Deep Belief Network and Back Propagation (DBN+BP) [24], Domain Adaptive Subspace Learning (DoSL) [14]. At the same time, PCA+SVM is selected as the reference algorithm for the experiment, and the SVM classifier adopts a linear kernel function. Table 4 shows the WAR results of the MTLSA and other advanced models and benchmark models in six cross-corpus recognition schemes.

It can be seen from Table 4 that the WAR of the proposed model MTLSA is higher than PCA+SVM, TSDSL and DBN+BP in six cross-corpus schemes, indicating that multi-task learning combined with subdomain adaptive reduction in feature distribution differences is advanced. Among them, TSDSL only reduces the feature distribution distance in the global domain emotion space, and ignores the connection between more fine-grained emotion categories, and the model in this chapter uses emotion labels and gender labels to divide the feature space into independent subdomain space, considering the confusing alignment influence of different emotion information, and accurately aligning the feature distribution of the same emotion and gender. DBN+BP belongs to the application of deep learning with the proposed model, but DBN+BP only uses the basic feature processing method, and does not use the correlation feature transfer learning algorithm to train the cross-corpus emotion classifier, so the cross-corpus recognition effect is not ideal, DoSL uses subspace learning methods, but only features reduction and dimension selection, and does not achieve accurate domain alignment. It is difficult to effectively improve the generalization of the cross-corpus speech emotion recognition model.

Compared with the above single task-learning method, the structure of multi-task learning is generally composed of shared modules and task modules. The shared modules contain shared network parameters, and the task modules contain different tasks that the network needs to complete. Multi-task learning trains multiple tasks in parallel by sharing network layer parameters, and finally enables a single network to achieve multiple functions, which is also the key to improving model generalization. It can be concluded that gender is an important factor affecting the performance of cross-corpus speech emotion recognition, and learning common gender information while extracting common emotion information can effectively alleviate the gender difference in emotional features and help further reduce the feature distribution distance between the source domain and the target domain.

## 6. Conclusions

This paper proposed a cross-corpus speech emotion recognition model based on multi-task learning and subdomain adaptation to alleviate the impact of gender factors on emotion recognition. The model takes emotion recognition as the main task, gender recognition as the auxiliary task, and uses the deep denoising auto-encoder as the shared network of the multi-task learning framework to extract the emotional common information and gender common information with strong representation ability. LMMD-based subdomain adaptive algorithm is used to constrain learning emotion and gender features, and further, obtain shared information. From a large number of experimental results, the model proposed in this chapter can not only effectively reduce the difference in feature distribution between the source domain and the target domain, but also alleviate the impact of gender attributes on emotion recognition, providing a new idea for solving the problem of cross-corpus speech emotion recognition.

## Figures and Tables

**Table 1:** Corpora information for cross-corpus identification.

Emotion Recognition Task			Gender Identification Task	
Corpus	Num of Samples	Emotional Tags	Male Samples	Female Samples
Berlin	375	Anger, Sad, Fear, Happy, Disgust	159	216
eNTERFACE	1072		885	187
CASIA	1000	Anger, Sad, Fear, Happy, Surprise	500	500
eNTERFACE	1072		847	225
Berlin	408	Anger, Sad, Fear, Happy, Neutral	187	221
CASIA	1000		500	500

**Table 2:** Six cross-corpus experimental schemes and identification tasks.

Scheme	Source Domain	Target Domain	Cross-Corpus Identification
E→B	eNTERFACE	Berlin	Anger, Sad, Fear, Happy, Disgust
B→E	Berlin	eNTERFACE	
E→C	eNTERFACE	CASIA	Anger, Sad, Fear, Happy, Surprise
C→E	CASIA	eNTERFACE	
B→C	Berlin	CASIA	Anger, Sad, Fear, Happy, Neutral
C→B	CASIA	Berlin	

**Table 3:** WAR of different ablation models in six cross-corpus schemes (%).

Model	E→B	B→E	E→C	C→E	B→C	C→B
MTLSA_L	36.80	24.44	32.90	23.23	30.10	39.95
MTLSA_M	55.73	30.60	34.40	30.32	39.30	53.94
MTLSA	**57.60**	**34.12**	**35.21**	**31.52**	**41.90**	**56.86**

**Table 4:** WAR of comparison model in six cross-corpus schemes (%).

Model	E→B	B→E	E→C	C→E	B→C	C→B	Average
PCA+SVM	50.85	33.68	28.60	27.80	33.60	43.87	36.40
TSDSL [22]	50.67	**35.47**	32.50	33.28	37.40	56.60	40.98
DBN+BP [24]	26.67	32.28	24.20	31.04	35.80	46.81	32.80
DoSL [14]	49.58	30.64	35.20	**33.90**	35.77	**57.51**	40.43
MTLSA	**57.60**	34.12	**35.21**	31.52	**41.90**	56.86	**42.87**

## Data Availability

Not applicable.
